# Multiple Mechanisms and Challenges for the Application of Allopolyploidy in Plants

**DOI:** 10.3390/ijms13078696

**Published:** 2012-07-13

**Authors:** Kenji Osabe, Takahiro Kawanabe, Taku Sasaki, Ryo Ishikawa, Keiichi Okazaki, Elizabeth S. Dennis, Tomohiko Kazama, Ryo Fujimoto

**Affiliations:** 1 Commonwealth Scientific and Industrial Research Organisation (CSIRO) Plant Industry, Canberra, ACT 2601, Australia; E-Mails: kenji.osabe@csiro.au (K.O.); liz.dennis@csiro.au (E.S.D.); 2 Watanabe Seed Co., Ltd, Machiyashiki, Misato-cho, Miyagi 987-8607, Japan; E-Mail: tkawa@beach.ocn.ne.jp; 3 Gregor Mendel Institute of Molecular Plant Biology, Austrian Academy of Sciences, Dr. Bohrgasse 3, Vienna 1030, Austria; E-Mail: taku.sasaki@gmi.oeaw.ac.at; 4 Laboratory of Plant Breeding, Graduate School of Agricultural Science, Kobe University, Nada, Kobe 657-8510, Japan; E-Mail: r-ishika@port.kobe-u.ac.jp; 5 Cell and Developmental Biology, John Innes Centre, Norwich Research Park, Colney, Norwich, NR4 7UH, UK; E-Mail: ryo.ishikawa@jic.ac.uk; 6 Graduate School of Science and Technology, Niigata University, Ikarashi-ninocho, Niigata 950-2181, Japan; E-Mail: okazaki@agr.niigata-u.ac.jp; 7 Graduate School of Agricultural Science, Tohoku University, Aoba-ku, Sendai 981-8555, Japan; E-Mail: tomo-kazama@bios.tohoku.ac.jp

**Keywords:** allopolyploid, self-compatibility, cytoplasmic male sterility, reproductive barrier, epigenetics

## Abstract

An allopolyploid is an individual having two or more complete sets of chromosomes derived from different species. Generation of allopolyploids might be rare because of the need to overcome limitations such as co-existing populations of parental lines, overcoming hybrid incompatibility, gametic non-reduction, and the requirement for chromosome doubling. However, allopolyploids are widely observed among plant species, so allopolyploids have succeeded in overcoming these limitations and may have a selective advantage. As techniques for making allopolyploids are developed, we can compare transcription, genome organization, and epigenetic modifications between synthesized allopolyploids and their direct parental lines or between several generations of allopolyploids. It has been suggested that divergence of transcription caused either genetically or epigenetically, which can contribute to plant phenotype, is important for the adaptation of allopolyploids.

## 1. Introduction

Polyploidization (whole genome duplication) is an important process in plant speciation [1]. Polyploidy is common in angiosperms, and in general there are two types of polyploids, autopolyploid (auto = same) and allopolyploid (allo = different). Autopolyploids are produced by multiplication of the genome from a single species. Allopolyploids are typically derived from hybridization between two (or more) distantly related species and combine divergent genomes with their own chromosome complements. Merging genomes from different species provides not only genome variation, but also novel opportunities to generate diversification through their interactions that allow allopolyploidy to function as a potential source of new species [1,2].

Several different pathways of allopolyploid formation have been described. The one-step model is that allotetraploids are formed by fusion of unreduced male and female gametes from two diploid species. Direct hybridization between two autopolyploid species is also categorized in a one-step model. The two-step model is that an allotetraploid is formed by an inter-specific cross between two diploid species followed by somatic doubling in meristematic tissues [3]. The triploid-bridge model is that triploids are formed by fusion of unreduced and reduced gametes from two diploid species and then unreduced gametes from triploids fuse with reduced gametes from diploids, which can generate stable allotetraploids [3,4].

Generation of natural allopolyploids may be sporadic because of limitations such as co-existing populations of parental lines, hybrid incompatibility (pre- and post-zygotic reproductive barriers), unreduced gametes, and chromosome doubling. However, allopolyploids are widely observed among plants and have been formed multiple times [5], indicating that allopolyploids succeed in breaking down hybrid incompatibility, forming unreduced gametes and undergo chromosome doubling during evolution. The most common route to allopolyploid formation is via unreduced gametes, which have been identified in many plant taxa [3]. Unreduced gametes are formed by meiotic dysfunction at the meiosis I (FDR: first division restitution) or at meiosis II (SDR: second division restitution). After successful generation of new allopolyploids, correct segregation of chromosomes is important for producing offspring as allopolyploids have more than two sets of chromosomes; suppression of crossovers between homoeologous chromosomes is required to ensure fertility. In allopolyploids, diploid-like meiotic behavior is observed, indicating that correct pairing of homologous chromosomes is controlled, in the case of wheat, the *ph1* locus performs this function [6].

Allopolyploids may have a selective advantage because of their common occurrence in nature. Heterosis, gene dosage, gene redundancy, and reproductive system advantages of allopolyploids have been suggested as the basis of this advantage [7,8]. Heterosis or hybrid vigour refers to the phenotypic superiority of a hybrid over its parents. Heterozygosity of the different genomes might induce growth vigour and become fixed in allopolyploids [9]. Several inter-specific hybrids show greater vigour than the average of their parental lines [9–11]. The hybrid between *Arabidopsis arenosa* and *Arabidopsis thaliana* shows a hybrid-vigour-like phenotype, and this phenomenon is considered to result from an alteration of circadian rhythm genes in hybrids [9,12]. The variation of dosage-regulated gene expression could be increased in allopolyploids, which may contribute to generating a new plant phenotype [8,13]. Gene redundancy has the potential to mask recessive deleterious alleles by dominant alleles [7]. In addition to genetic robustness against null mutations, it has been suggested that duplicated genes produce a diversity of expression that facilitates adaptive evolution [14]. Another advantage of allopolyploids is their reproductive system. Most allopolyploids show self-compatibility that can favor rapid reproduction, while some of their parents are self-incompatible [7,15]. Although self-compatibility has the potential to lead to inbreeding depression by the accumulation of recessive alleles, allopolyploids are more tolerant than diploids to this because of their genome redundancy.

Allopolyploids include important crops such as wheat, cotton, and canola, and all have improved agricultural traits relative to their diploid progenitors. Wheat was domesticated about 10,000 years ago, and bread wheat, *Triticum aestivum*, has a genome composition of AABBDD, which arose by inter-specific hybridization between *T. turgidum* (AABB) and *Aegilops tauschii* (DD) [16,17]. Durum (pasta) wheat has a genome composition of AABB. In cotton (Gossypium), there are two major branches of Gossypium diploid species, the New World (D-genome group) and the Old World (A, G, *etc*.). The commercially important cottons are naturally formed allopolyploids (AADD) such as *Gossypium hirsutum* (Upland cotton) and *G. barbadense* (Pima or Egyptian cotton), and they have superior fiber yield and quality relative to their ancestral species [18,19]. Canola (*Brassica napus*) is an allopolyploid (AACC), with the A and C genomes corresponding to the genomes of *Brassica rapa* and *Brassica oleracea*, respectively [20]. *B. rapa* (Chinese cabbage, turnip, *etc*.) and *B. oleracea* (cabbage, broccoli, *etc*.) are cultivated mainly as vegetables, while canola is cultivated as an oil crop. In this way, allopolyploidy has played a crucial role in the domestication of crops and their selection for desirable products [16].

Artificially synthesized allopolyploids are excellent material to understand early events following hybridization because the exact progenitors are known. Methods for synthesizing allopolyploids have been developed and artificially synthesized allopolyploids have been widely generated. Molecular studies using artificially synthesized allopolyploids have revealed genomic rearrangements [21–23], DNA methylation alterations [24–26], transposon activation [27–29], and transcriptome changes [11,30–36]. Transcriptome changes caused genetically or epigenetically (transcriptional states of genes can be controlled by changes to the structure of chromatin without any change in the DNA sequence) have the potential to provide a source of developmental novelty, and might be important for the evolutionary success of allopolyploids.

## 2. Techniques for Synthesizing Allopolyploids

**Figure 1:**
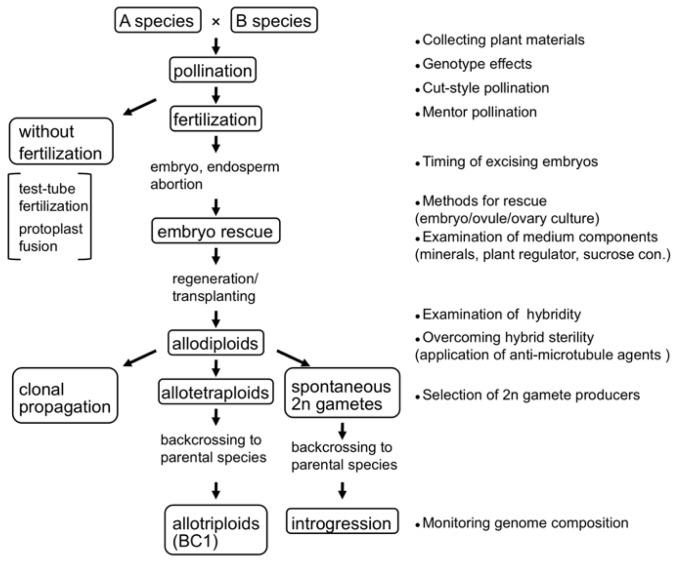
Procedures and technical remarks of making synthetic allopolyploids.

In order to successfully produce synthetic allopolyploids, various factors related to inter-specific hybridization and subsequent procedures should be considered (Figure 1). As the level of cross-ability varies between parental materials, various combinations of genotypes have been tested in inter-specific breeding programs of many crops [37–39]. Whether a genotype is used as the maternal or paternal parent is important in inter-specific hybridization because unilateral incompatibility is often observed, especially in crossing combinations between self-incompatible and self-compatible species [40]. In lilies, crosses between remotely related species succeeded in only one direction [41]. Environmental factors such as temperature can also influence cross-ability: seed set was promoted at 15 °C in the inter-specific crosses of tulips [42], and at 23–26 °C in the crosses between *Cucumis sativus* and *C. metuliferus* [43]. The production of synthetic allopolyploids requires knowledge about the cross-ability of the species and sensitive techniques to overcome the limitations of incompatibility.

In lilies, the pollen tubes arrest halfway down the stylar canal after inter-specific pollination (post-pollination pre-zygotic barriers), but this can be efficiently overcome by placing pollen on the cut-style [44,45]. This technique led to the generation of new hybrid lilies such as LA (between *L. longiflorum* and Asiatic hybrids) hybrids, which were distributed commercially. In earlier studies, mentor pollen techniques were successfully applied to overcome pre-zygotic barriers in *Populus* [46], apple [47], *Cucumis* [48], and pear [49]. Application of plant growth regulators to ovaries at the time of pollination improved seed set after inter-specific hybridization of *Lilium* [50]. Applications of these techniques are species specific, and further studies are required for bypassing pre-zygotic barriers.

Embryo rescue including embryo, ovule, and ovary culture is necessary to produce hybrids between distantly related species where a post-zygotic barrier is caused by insufficient endosperm formation (Chapter 3). For successful embryo rescue, it is necessary to first determine the optimal isolation time of immature hybrid embryos (days after pollination). In the case of a cross between *B. oleracea* and *B. rapa*, this timing was 20–30 days after pollination. *In vitro* culture conditions of embryos has been extensively discussed [51,52] and various culture procedures have been applied to produce inter-specific hybrids. For instance, the culture of ovules obtained from the cross between *Nicotiana tabacum* and *N. acuminata* [53] was conducted using liquid Nitsch H medium containing 5% sucrose. The hybrid embryos obtained from the cross between *B. oleracea* var. *alboglabra* and *Brassica campestris* were cultured on White’s medium, containing 0.8% agar, 4% sucrose, and 10% coconut milk [54]. Asano and Myodo [55], working with *Lilium* embryos derived from the cross of distantly related species, showed that Murashige and Skoog medium is suitable for the culture of immature embryos when it was adjusted to pH 5.0 and supplemented with 20–40 g/L sucrose and 10^−4^–10^−2^ mg/L NAA (Naphthalene acetic acid). Okazaki *et al*. [56] revised the optimum sucrose concentration to 6% for embryo culture of lilies.

Hybrids obtained from distantly related inter-specific crosses are mostly sterile and cannot be used in cross breeding [57–59]. Restoration of hybrid fertility can be achieved by zygotic or somatic chromosome doubling and by gamete non-reduction (unreduced gamete) [60–63]. The anti-microtubule agent, colchicine, is effective for somatic (mitotic) polyploidization [64]. Dinitroaniline herbicides such as trifluralin, oryzalin, or amiprophosmethyl also act as anti-microtubule agents and they are applied at a lower concentration (0.001–0.01%) compared to colchicine treatment (0.05–0.2%) [63–68]. Oryzalin was more efficient for inducing chromosome doubling and less toxic than colchicine [65,68]. Various methods of application of colchicine were adopted: treating flower buds under vacuum, soaking plantlets with colchicine solution, dropping colchicine on to the apical meristem, applying it to the leaf axils with cotton wool, *etc*. [64]. Nitrous oxide (N_2_O) has been applied to zygotes [69], seedlings [70], pollen mother cells [71,72], and anther somatic cells [73], as a polyploidizing agent in lieu of colchicine treatment. Nitrous oxide may act through depolymerization of microtubules [74]. Nitrous oxide is suitable for treating organs inside tissues because the gas can permeate to reach the tissues of interest, e.g., developing microspores within tulip bulbs [71]. Additionally, the gas is expected to be rapidly dissipated from treated tissues when the pressure is released, thereby preventing further harmful after-effects. For these reason nitrous oxide has been applied as a polyploidizing agent to overcome hybrid sterility in lilies [73,75].

Somatic chromosome doubling of a diploid inter-specific hybrid produces an allotetraploid containing two diploid genomes from two different species. In allotetraploids, the homologous chromosome from each parental species pairs during meiosis and leads to normal meiosis and fertile gamete production [60,62,63,76]. At the same time, such a preferential homologous chromosome pairing has the drawback of inhibiting meiotic recombination between genomes coming from different progenitors. The resulting fertile gametes show a few variations, so that the progeny of allotetraploids exhibit fixed heterozygosity [61,77–79]. By contrast, spontaneous formation of unreduced gametes from aberrant meiosis (sexual polyploidization), *i.e.*, FDR or SDR, occurs in diploid species as well as allodiploids [61,79]. Lim *et al*. [80] reported a new type of 2n gamete formation mechanism, IMR (indeterminate meiotic restitution), which combines characteristics of FDR and SDR: during the first meiotic division, some of the univalents divide equationally (as in FDR) and some bivalents disjoin reductionally (as in SDR) before telophase, leading to a dyad without further division. Such 2n-gametes can be used for the production of sexual progeny either through crossing or selfing [62,80–84]. In addition, sexual polyploidization forces homoeologous chromosome pairing during meiosis in allodiploids, promoting genetic recombination between homoeologous chromosomes. Therefore, unlike in somatically doubled allotetraploids, fertile pollen derived from sexual polyploidization can transmit huge genetic variation to the progeny, which is preferable for introgression breeding via backcrossing with diploid parental species [78,79,84].

## 3. Reproductive Barrier

There are many reproductive barriers to the natural formation of allopolyploids at pre- and post-zygotic stages [85]. Pre-zygotic barriers include geographic or pollinator isolation (*i.e.*, flower structure or color) [85] and flowering time [85,86]. The inhibition of pollen tube germination, arrest of pollen tube growth in ovules, and unilateral incompatibility (asymmetric patterns of pollen rejection in crosses between different species) are involved in post-pollination pre-zygotic barriers [85]. Once the pollen-tube germination and fertilization have been successful, there are still reproductive barriers such as hybrid inviability, a post-zygotic barrier [85]. Under- or over-proliferation of endosperm, which supports embryo development by providing nutrients crucial for viable seed formation, is one post zygotic barrier, which depends on the crossing combination [87–90]. A common characteristic of unusual endosperm development observed in inter-specific and inter-ploidy crosses is due to a premature or delayed cellularization. Recent studies suggested that the altered development of endosperm is due to dysregulation of genomic imprinting [91]. Another post-zygotic barrier is hybrid sterility caused by sets of interacting genes and is described as the Dobzhansky-Muller incompatibility model [85]. Sometimes inter-specific hybrids show male-sterility, even though they have succeeded in polyploidization. One type of male-sterility is caused by incompatibility between the nuclear genome and the maternally derived mitochondrial genome, termed cytoplasmic male sterility (CMS) [92]. Inter-specific and inter-generic crosses sometimes lead to CMS, because a change of nuclear component has impacts on mitochondrial gene expression [93]. In this chapter, we introduce genomic imprinting and CMS in detail as their molecular mechanisms have recently become clear. The understanding of imprinting and CMS will enable us to generate allopolyploids and inter-specific hybrids efficiently.

### 3.1. Genomic Imprinting

**Figure 2:**
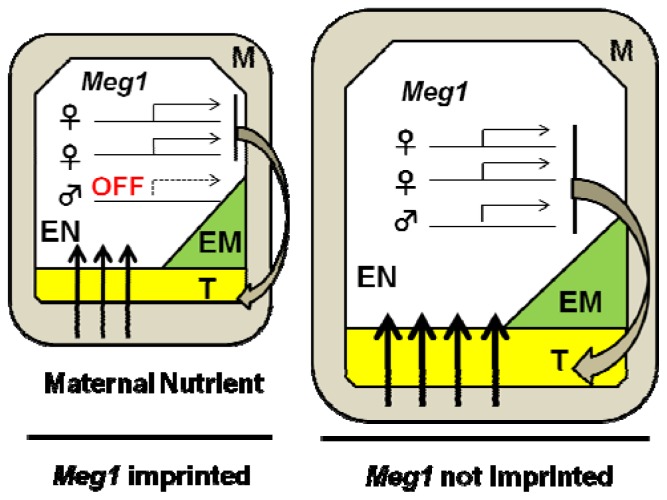
Imprinting of maize *Meg1* gene and its role on maternal nutrient allocation. *Meg1* imprinting limits the differentiation of transfer tissue and therefore nutrient allocation and seed size is affected when regulation of imprinting is disrupted. Nutrient allocation is shown by the size of arrows. T: Transfer tissue, EM: Embryo, EN: Endosperm, M: Maternal tissue.

In general, maternally and paternally inherited alleles of each gene are expressed equivalently in diploids but, in some genes, the two alleles are expressed at different levels depending on their parent of origin, termed genomic imprinting. Genomic imprinting is observed in both mammals and flowering plants and is regulated by DNA methylation or histone modifications. Genes involved in genomic imprinting have been extensively studied using the model plant, *A. thaliana* [94]. Analyses of mutants showing a prolonged syncytial phase and delayed endosperm cellularization identified genes encoding the components of a Polycomb complex [94]. In endosperm, only the maternal alleles of *MEA* (*MEDEA*) and *FIS2* (*FERTILIZATION INDEPENDENT SEED 2*) are expressed, while the paternal alleles are silenced [95,96]. On the other hand, *PHE1* (*PHERES 1*), a gene identified as a downstream target of the Polycomb complex, was expressed from the paternal allele [97]. Imprinting of these genes is regulated by differences in genomic DNA methylation patterns between the embryo and endosperm [94]. *MEA* and *FIS2* are maternally expressed while *PHE1* is paternally expressed, and they regulate endosperm development in opposite directions. This antagonistic regulation may explain the observed phenotypes of inter-specific crosses, and is consistent with the parental conflict hypothesis [98]. Recently, *Meg1* (*Maternally expressed gene 1*) in maize has been shown to be required for the development of the endosperm nutrient transfer cells between seed and maternal tissue. *Meg1* also plays important roles in the regulation of maternal nutrient uptake and sucrose partitioning. This result demonstrated the first functional evidence that an imprinted gene is involved in a balanced distribution of maternal nutrients to filial tissues in plants, although *Meg1* is a maternally expressed imprinted gene that promotes rather than restricts nutrient allocation (Figure 2) [99].

Several studies have indicated the involvement of genomic imprinting in reproductive barriers. The hybrid endosperm derived from *A. thaliana* as the female parent and its close relative, *A. arenosa* as the male parent, shows overgrowth with altered or arrested embryo development [100]. The imprinted gene expression patterns of *MEA* and *PHE1* were disrupted and bi-allelic expression was detected in the hybrid endosperm. The overgrowth phenotype of endosperm is probably caused by the maternal de-repression of *PHE1*, suggested by the findings that the maternal *phe1* mutation improves fertility [100–102]. Disruption of *PHE1* imprinting may cause a prolonged and stable interaction with *AGL62* (*AGAMOUS-LIKE 62*), which inhibits cellularization [103,104]. Altered expression of the *PHE1* homolog, *OsMADS87*, is observed in the endosperm of hybrids between cultivated and wild rice species [105]. These findings suggest that disruption of the regulation of genomic imprinting may be caused by the difference of epigenetic modifications on *PHE1* between species, and it may act as a reproductive barrier in species hybridization and allopolyploidization. This idea also supports the ‘Endosperm balance number (EBN) hypothesis’, in which the ratio 2:1 of maternal to paternal EBN value is important for a successful cross [88–90]. Analysis of genomic imprinting in allopolyploids will provide important findings on its role in species hybridization.

### 3.2. Cytoplasmic Male Sterility

Inter-specific hybrids sometimes show male-sterility, which is known as hybrid sterility. Occasionally male sterility is caused by incompatibility between mitochondrial and nuclear genomes (alloplasmic male sterility), a type of CMS (Cytoplasmic male sterility). CMS phenotypes range from floral abnormalities to failure of pollen maturation. CMS is often observed in natural plant populations, and can also be artificially produced by successive backcrossing resulting from inter-specific exchange of nuclear and cytoplasmic genomes. CMS is a maternally inherited phenotype and known to be governed by the mitochondrial genome. Plant mitochondria are quite different from those of animal mitochondria: the animal mitochondrial genome is less than 20 kb in size and has a circular structure, while plant mitochondrial genomes are large (200 to more than 2400 kb; dependent on species) and complex having a multipartite structure because of large repetitive sequences [106]. Such repetitive sequences in plant mitochondrial DNA can induce recombination, and create chimeric *orf*s (*open reading frames*) [92]. Expression of certain new *orfs* is associated with CMS. From a comparison of mitochondrial genomes or gene expression between CMS and normal cytoplasms, CMS-associated genes have been reported in many species [107]. These genes often show a chimeric structure with the mitochondrial genes in normal mitochondria and have transmembrane domains. Expression of the CMS-associated gene is occasionally suppressed by a particular nuclear factor being transported into mitochondria, and results in fertility restoration. A gene for this nuclear factor is called *Rf* (*Restorer of fertility*).

*Rf* genes are grouped into two types, PPR (pentatricopeptide repeat) type and non-PPR type. The PPR proteins are nuclear-encoded RNA binding proteins, which function in post-transcriptional processes (RNA editing, RNA splicing, RNA cleavage and translation) in organelles [108]. *Rf* genes in *Petunia hybrida* (*Rf-PPR592*), *Raphanus sativus* (*Rfo*/*Rfk*), *Oryza sativa* L. (*Rf1a* and *Rf1b*) and *Sorghum bicolor* (*Rf1*) encode PPR proteins [109–114]. These PPR type RF proteins have been investigated and reported to have roles in decreasing CMS-associated gene products in each mitochondrion [115–117]. These analyses suggest that PPR type RF proteins are key factors that directly eliminate causes of CMS in mitochondria. The non-PPR type *Rf* genes include *Rf2a* of maize (*Zea mays*), which encodes aldehyde dehydrogenase, and *Rf2* and *Rf17* of rice (*O. sativa*), which encode glycine-rich and ACPS-like (acyl-carrier protein synthesis-like) domain containing proteins, respectively [118–120]. RF2a in maize does not reduce the accumulation of a CMS-associated gene product, URF13, although RF2a is required for normal anther development [121]. The presence of *Rf17* in rice also does not affect the RNA profile of a CMS-associated gene, CW-*orf307* [122]. The reduced expression of *Rf17* restores the fertility of CW-type CMS rice. The expression of *Rf17* is dependent on the mitochondrial haplotypes [119], indicating that signals from the mitochondria to the nucleus (retrograde signals) control the expression of *Rf17*.

**Figure 3:**
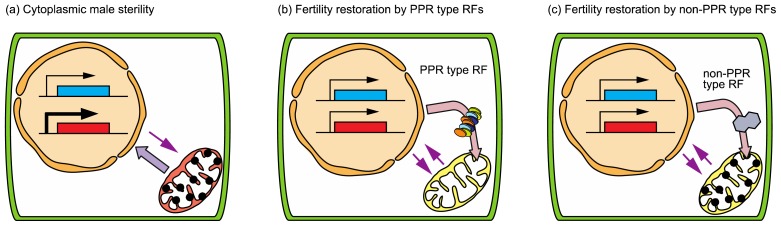
Hypothetical retrograde signals from mitochondria involved in cytoplasmic male sterility (CMS)/restoration of fertility systems. (**a**) Retrograde signals from CMS mitochondria, which are enhanced by CMS-associated gene products, disturb the expression of a certain nuclear-encoded gene essential for pollen development. Such imbalance leads male sterility. (**b**) A PPR-type RF protein is imported into mitochondria and suppresses the expression of the CMS-associated gene and recovers the mitochondrial state to normal, which reduces the retrograde signals and restores the expression of a nuclear-encoded gene essential for pollen development. (**c**) Non-PPR type RF protein is imported into mitochondria and functions to improve the mitochondrial metabolic state. Although the CMS associated gene products still exist in mitochondria, retrograde signals and fertility are restored.

Retrograde signals from mitochondria determine whether accumulation of CMS-associated gene products in mitochondria leads to male sterility [123]. It is hypothesized that CMS is caused by insufficient ATP-production for pollen development due to the toxic effects of CMS-associated gene products in mitochondria. A new hypothesis is suggested from the point of view of retrograde signaling as shown in Figure 3. CMS associated gene products are accumulated in CMS mitochondria, which leads to an abnormal mitochondrial state and triggers emission of enhanced retrograde signals to disturb the expression of nuclear-encoded genes essential for pollen development. Such imbalance of nuclear gene expression during anther development leads to male sterility (Figure 3a). When a PPR type *Rf* gene exists in the nuclear genome, the RF protein is transported into mitochondria and suppresses expression and/or accumulation of CMS-associated gene products. As a result of RF function, the CMS mitochondria recover and reduce retrograde signals, so that the expression pattern of nuclear-encoded genes becomes identical to that of fertile plants with normal mitochondria, resulting in fertility restoration (Figure 3b). In the case of fertility restoration by non-PPR type RF proteins, the accumulation of the CMS-associated gene products is not changed. The RF protein may act to improve the metabolic state of CMS mitochondria, resulting in a bypass for fertility restoration (Figure 3c). Studies on the molecular entity of retrograde signals from CMS mitochondria will elucidate the details of CMS/*Rf* systems. This knowledge will help us to avoid hybrid sterility caused by incompatibility between mitochondrial and nuclear genomes when generating inter-specific hybrids.

## 4. Self-Compatibility

Self-incompatibility is a mechanism for preventing self-fertilization in many plant species. Self-incompatibility recognition specificity of stigma and pollen is controlled by a single multi allelic locus, called an *S* locus. There are two major classes of self-incompatibility, gametophytic and sporophytic. In gametophytic self-incompatibility, the *S* phenotype of pollen is determined by its own haploid genome, while in sporophytic self-incompatibility the *S* phenotype of pollen is determined by the parental diploid genome [124].

Brassicaceae species have sporophytic self-incompatibility, and the self-incompatibility response is based on the *S* allele specific interaction between the stigma determinant, SRK (*S* receptor kinase), and the pollen determinant, SP11/SCR (*S*-locus protein 11/*S*-locus cysteine-rich protein) (hereafter called SP11): SRK and SP11 derived from the same *S* alleles bind to each other, and this interaction triggers inhibition of pollen tube germination or elongation [124,125]. These two genes, *SRK* and *SP11*, are normally not separable by recombination and are transmitted to progeny as one set, termed *S* haplotypes [124,125]. There are dominant relationships of *S* haplotypes in the pollen of *S* heterozygous plants (class-I; dominant, class-II, recessive) [125]. Transcription of class-II *SP11* genes is suppressed in the pollen of the class-I/class-II *S* heterozygous plants [125], and this suppression is associated with stage and tissue specific *de novo* DNA methylation in the promoter regions of class-II *S* haplotypes [126]. Recently, Tarutani *et al*. [127] showed that small RNAs, *Smi* (*SP11 methylation inducer*), are expressed from the *S* locus of class-I *S* haplotypes, and are homologous to the promoter region of class-II *S* haplotypes. The *Smi* RNAs can drive the *de novo* DNA methylation of the promoter region of the class-II *SP11* allele leading to suppression of its expression in the pollen of the class-I/class-II *S* heterozygous plants [127].

**Figure 4:**
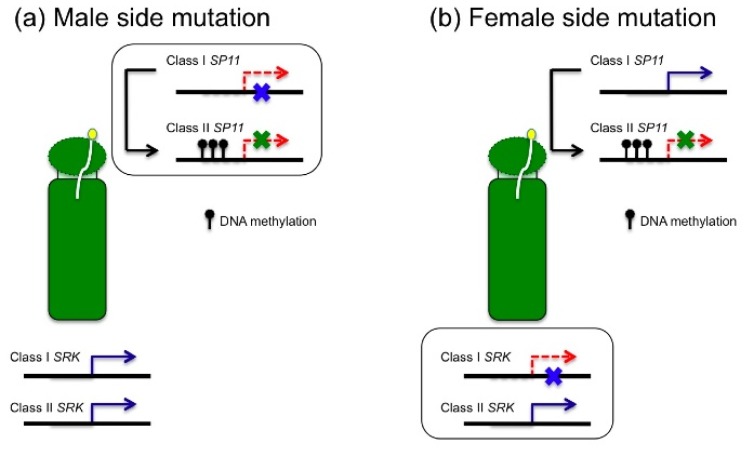
Two types of mutation cause self-compatibility in *B. napus*. (**a**) Class-I SP11 lost its function by mutation (blue cross), while expression of class-II *SP11* is suppressed by dominant relationship in pollen (green cross). (**b**) Class-I SRK lost its function by mutation (blue cross), and class-II *SP11* is silenced by dominant relationship (green cross).

Okamoto *et al*. [15] suggested that the dominant relationship mentioned above is important for self-compatibility of *B. napus*. As *B. napus* is an allotetraploid having an AC genome, it has *S* loci derived from both the A and C genomes. Most *B. napus* have both class-I and class-II *S* haplotypes (one is derived from A genome and the other is from C genome), and class-I *S* haplotypes have lost the function of SP11 or SRK by spontaneous mutations. Complementation experiments confirmed that loss of function in class-I *SP11* by mutations caused self-compatibility in ‘Westar’ of *B. napus* [128]. In the artificially synthesized inter-specific hybrid between *B. rapa* with class-I *S* homozygous alleles and *B. oleracea* with class-II *S* homozygous alleles, expression of *SP11* in class-II *S* haplotype was suppressed, indicating that there is a dominant relationship occurred between different chromosomes [129]. So when class-I SRK lost its function by mutation, *B. napus* became self-compatible because the expression of class-II *SP11* was suppressed by a dominant relationship (Figure 4) [15]. When class-I *SP11* lost its function by mutation, expression of class-II *SP11* was still suppressed by the dominant relationship (Figure 4) [15], as the expression of class-I *SP11* is not essential for suppression of class-II *SP11* in the *S* heterozygotes [130]. Mutations occurring in the *S* determinant genes (*SRK* or *SP11*) of both A and C genome alleles at the same time is highly unlikely. However, if there is a dominant relationship between *S* haplotypes of the A and C genome, a mutation of only a dominant *S* haplotype is sufficient for loss of self-incompatibility. Thus a dominant relationship may be utilized for generating self-compatible allopolyploids in the process of evolution.

In addition to the genetic mutations described above, the possibility that epigenetic change contributes to generating self-compatibility of allopolyploids has been suggested [131]. Although an inter-specific hybrid between *A. thaliana* and *A. lyrata* has never been detected in nature, this inter-specific hybrid was obtained by crossing between self-compatible *A. thaliana* as a female parent and self-incompatible *A. lyrata* as a male parent [10,11]. In the stigma of the hybrid, the expression of *SRK* derived from *A. lyrata* was reduced, and became compatible with the pollen of parental *A. lyrata* [131]. The stigma of BC_1_F_1_ (backcrossed *A. lyrata* to inter-specific F_1_ hybrid between *A. thaliana* and *A. lyrata*) showed a self-incompatibility response, suggesting that the disruption of the expression pattern of *SRK* in inter-specific hybrids, which leads to self-compatibility, may be due to the epigenetic changes [131]. As several studies have reported that there are widespread changes to gene expression between hybrids and their progenitors (chapter 6), *de novo* changes in expression of self-incompatibility recognition specificity genes might be important for self-compatibility.

Out-crossing enforced by self-incompatibility may play a role in the avoidance of inbreeding depression, and self-incompatibility is advantageous in the process of evolution over the long term [7,132]. When there is mate limitation, self-compatibility rather than self-incompatibility is advantageous in the short-term (reproductive assurance) [132]. Allopolyploids are generally self-compatible, though their putative parental lines are self-incompatible [15]. Synthesized inter-specific hybrids between self-incompatible parental lines are self-incompatible [15,129], suggesting that allopolyploids lose self-incompatibility. Natural formation of allopolyploids may be uncommon, and self-compatibility can be advantageous for species survival when mating partners are limited: self-compatibility can establish new populations from individual plants [132]. Moreover, as allopolyploids have more than two different genomes (heterozygosity), the risk of inbreeding depression is smaller than in diploid plants and reduces the selective pressure to maintain out-crossing [7]. It can be speculated that self-compatibility has been selected in allopolyploids during evolution.

## 5. Genetic and Epigenetic Changes

Synthetic allopolyploid plants can readily provide material to investigate the changes that occur immediately after polyploidization and through subsequent generations. Unlike natural allopolyploids, the parents can be selected, and this allows comparison between the parents and the synthesized allopolyploids. Evidence of genomic changes has been demonstrated in synthesized allopolyploids such as wheat, *B. napus*, cotton, and *Arabidopsis suecica*, and there are variable levels of genomic changes among different allopolyploids. In synthesized wheat, genomic elimination occurred shortly after allopolyploid formation [133]. By contrast, there is no genomic elimination or rearrangement in euploid plants of wheat allohexaploids [134]. Most of the synthesized allohexaploids exhibit homologous pairing at metaphase I, but some aneuploids were observed especially in S0 generations, which are dependent on the progenitor combinations [134]. No homoeologous pairing is observed in synthesized wheat, which might be due to the function of the *Ph1* gene [134]. In synthesized *B. napus*, genetic changes are rare at the S0 generation [25,135], but genetic changes are much more frequent in the S5 generations [22]. Phenotypic variation increased in the following generations and was associated with DNA fragment losses due to homoeologous chromosome rearrangements, suggesting that chromosomal rearrangements may play a role in phenotypic variation [22]. In synthesized *B. napus*, homoeologous pairing, aneuploidy, and homoeologous chromosome compensation are observed, and pollen viability is negatively correlated with increasing aneuploidy [136–138]. Though there is variation in the level of crossover suppression between homoeologous chromosomes in natural *B. napus*, natural *B. napus* shows stronger control over homoeologous paring than synthesized *B. napus* [136,139]. A low level of genomic changes was detected in synthesized *A. suecica*. Synthetic *A. suecica* are meiotically stable and the frequencies of aneuploidy and chromosome abnormalities are relatively low [28], but rapid rearrangements in some specific regions such as rDNA loci were detected [140].

In addition to genetic changes, epigenetic modifications such as DNA methylation or histone modification are also affected in allopolyploids, probably caused by “genome shock” as McClintock predicted [21,141–143], and recent studies indicate that small RNAs have diverse roles in allopolyploid formation. Some transposons were transcriptionally activated or transposed in hypo-methylated mutants [144–147]. Transcriptional activation in specific transposons occurred in allopolyploids, which might be caused by “genome shock” [23,28,141]. Microarray analyses indicated that genome-wide transposon activation did not occur in inter-specific hybrids between *A. thaliana* and *A. arenosa* or between *A. thaliana* and *A. lyrata* [11,33].

One of the best known epigenetic phenomena observed in allopolyploids is nucleolar dominance, a phenomenon where one parental set of nucleolar rRNA genes is epigenetically silenced in the hybrids or allopolyploids, and the direction of silencing is consistent [148]. This phenomenon is observed in many organisms such as plants, fruit flies, frogs, and mammals. In inter-specific hybrids between *A. thaliana* and *A. arenosa* or between *A. thaliana* and *A. lyrata*, the *A. thaliana*-derived rRNA gene is silenced [11,26,149,150]. The involvement of epigenetic regulation is indicated by the de-repression of silencing by treatment with the DNA methyltransferase inhibitor 5-azaC (5 azacytidine) or the histone deacetylase inhibitor, TSA (trichostatin A), in Arabidopsis, Brassica, and Triticale hybrids [151,152]. Treatment with both 5-azaC and TSA showed no additional effects, indicating DNA methylation and histone deacetylation work in the same pathway [152]. The silenced *A. thaliana*-derived rRNA gene is enriched with repressive epigenetic marks such as DNA methylation and H3K9 di-methylation, whereas the active *A. arenosa*-derived gene is enriched with active epigenetic marks like H3K4 tri-methylation [153]. This epigenetic silencing is established during early postembryonic growth, and the *A. thaliana*-derived gene is progressively associated with silent modifications [154]. Factors required for nucleolar dominance, such as histone deacetylase; HDA6 (Histone deacetylase 6) and HDT1 (Histone deacetylase 1), *de novo* DNA methyltransferase DRM2 (Domains rearranged methyltransferase 2), RNA-directed DNA methylation pathway components; RDR2 (RNA-dependent RNA polymerase 2) and DCL3 (Dicer-like 3), and methylcytosine binding proteins; MBD6 (Methyl-CpG-binding domain 6) and MBD10, were identified from RNAi screening [153,155,156]. Knockdown of these genes showed de-repression of silencing of the *A. thaliana*-derived rRNA gene, and this activation was associated with increased active histone modification of the *A. thaliana*-derived copy.

**Figure 5:**
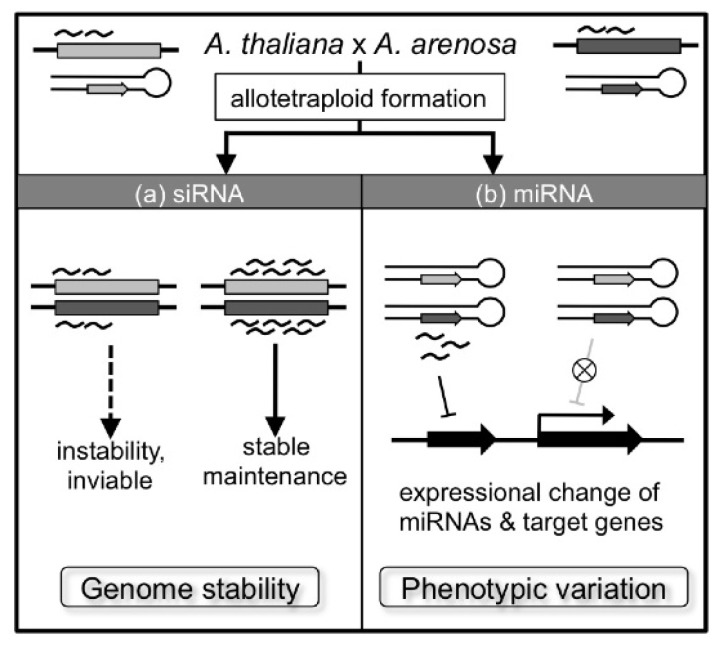
Roles of small RNA in allotetraploid formation. In allotetraploid formation, expression of small RNAs is changed. (**a**) Reduced repeat-associated siRNA would cause genomic instability and such re-synthesized F_1_ would be unviable. On the other hand, F_1_ with enriched siRNA would form stable allotetraploid. (**b**) Changes of miRNAs expression in F_1_ would induce diversity of the accumulation of target mRNAs. These transcriptional changes would induce phenotypic diversity in allotetraploid [157].

Small RNAs may have important roles in allotetraploid formation, as changes in expression/accumulation of small RNAs are observed in inter-specific hybrids [157,158]. Ha *et al*. [157] analyzed small RNA accumulation in *A. thaliana*, *A. arenosa*, a natural allotetraploid *A. suecica*, and a re-synthesized allotetraploid between *A. thaliana* and *A. arenosa* (F_1_ and F_7_ plants). *A. thaliana*-derived repeat- and transposon-associated siRNAs (small interfering RNAs), which are important for silencing of these sequences, were reduced in F_1_, but many siRNAs that disappeared in F_1_ were restored in F_7_ and their accumulation was similar to *A. suecica* [157]. These data suggest that repeat-associated siRNA functions as a genetic buffer in allotetraploid formation: reduced repeat-associated siRNA causes instability of re-synthesized allotetraploid plants, and siRNA-maintained plants form stable allotetraploid plants (Figure 5a) [157]. Expression of other classes of small RNAs, such as miRNAs (microRNA) and tasiRNAs (*trans*-acting siRNA), are changed as well in the inter-specific hybrid between *A. thaliana* and *A. arenosa*, and in the natural allotetraploid *A. suecica*. Many miRNA-targeted genes are differentially expressed in allotetraploids, indicating that a change of miRNA expression would induce phenotypic diversity in allotetraploids (Figure 5b) [157]. In fact for example, Ng *et al*. [159] suggest that the changed expression of miR163 induces variation in defense response by changing the amount of target transcripts of miR163 and secondary metabolites in allotetraploids.

## 6. Changes in Homoeologous Gene Expression

Some crops are allopolyploids, and it is important to understand the interaction and contribution of the homoeologs to the phenotype, and apply the knowledge for potential agronomical improvements. Allopolyploid formation is generally accompanied by gene expression change and, in synthetic wheat allohexaploids, the homoeologous gene expression changes established in early generations are similar to those in the natural allohexaploid [160]. Thus, it is important to understand the processes involved in the immediate genomic changes that occur in allopolyploids, not only to apply the knowledge for improvement of recently or artificially formed allopolyploid crops but to understand the evolutionary history of ancient allopolyploids.

Gene expression analyses between parental lines and synthetic allopolyploids have revealed changes in the context of additive (gene expression being equal to the average of the parental gene expression level) and non-additive (gene expression being different to the average of the parental gene expression level) gene expression, and the homoeologous expression changes can be meta-stable or stably inherited over generations. One of the interesting gene expression changes observed in allopolyploids is parental-biased gene expression. Parental-biased gene expression change refers to biased expression of homoeologous genes (up- or down-regulated) depending on the parent-of-origin. Strong parental-biased expression has been reported in newly synthesized allohexaploid wheat [21,30,35,161,162] and allotetraploid cotton [36,163–166]. In allotetraploid cotton (AADD, *G. hirsutum*) fiber cells, 30% of homoeolog expression was biased to either the A- or the D-ancestral genome [163]. Biased homoeolog expression in cotton has also been shown by microarray [36,164] and there are evidences for biased homoeolog expression of the D-genome over the A-genome [163,165,166]. Biased homoeologous expression has been shown in other allopolyploid species such as Arabidopsis, wheat, sugarcane, and *C x hytivus* [21,28,167,168]. Organ-specific and biotic/abiotic stress-related bias of homoeologous expression has been observed in allotetraploid cotton [31,169,170], *Nicotiana* [171], and Tragopogon [172], demonstrating that polyploid genome expression can be flexible, even soon after polyploid formation.

Change in homoeolog expression is expected to affect protein expression, and ultimately phenotype. Study on re-synthesized allotetraploid *B. napus* revealed that 2/3 of the investigated additive homoeologs exhibited non-additive protein expression [173]. Another study on synthesized *B. napus* followed protein expression changes across four generations (F_1_–F_4_) and reported stochastic changes in protein level across generations that were silenced in one generation and reactivated in another [174]. These stochastic changes suggest a potential relationship between small RNAs that are involved in epigenetic regulation of genes at early generations of allopolyploid formation, and translational regulation of proteins by small RNAs.

In the long-term, stably inherited homoeologous genes can experience sub-functionalization (if the homoeologs complement each other to retain the original gene function), neo-functionalization (acquiring new function), or pseudo-functionalization (homoeolog loses its original function) [1]. These evolutionary fates of homoeologous genes are gradual processes that follow immediate genomic changes after an allopolyploidization event. In *A. thaliana x A. arenosa* hybrid plants, 0.4% of genes were silenced immediately after polyploidization, but expression variation between *A. thaliana* and *A. arenosa* that diverged 1.5 million years ago, show 2.5% gene expression difference [28]. In newly synthesized allohexaploid wheat and allotetraploid cotton, 5% of genes are silenced within several generations after polyploidization [21,30,31]. In natural allotetraploid cotton, which has experienced polyploidization 1–2 million years ago, 25% of assessed homoeologous genes were silenced or biased in their expression [169]. In a recently formed allopolyploid species, Tragopogon, possible sub-functionalization was reported occurring within 80 years (or 40 generations) [172]. A recent study of gene expression changes in allohexaploid wheat suggests that half of the duplicated genes are structurally (chromosomal rearrangements) and functionally (sub-functionalization and neo-functionalization) altered within 10 million years, and the pseudo-functionalization process is completed within 45–50 million years [175]. Rice is another plant that has experienced polyploidization events [176], and expression divergence between paleoduplicates of rice, has shown 88–96% gene expression divergence within 50–70 million years [177]. The degree and speed of homoeolog expression divergence after polyploidization can vary depending on the species, but it is evident that homoeologous expression divergence occurs immediately after polyploidization and gene silencing can continue through evolution.

## 7. Conclusion

It is indubitable that allopolyploids have selective advantages. Allopolyploidy can cause dynamic changes in gene expression due to functional homoeologous copies interacting with each other, to changes in selective pressure on one of the homoeologous copies, and/or to epigenetic changes, which require orchestration of the genes for the plant to survive the “genome shock”. Interaction of homoeologs plays a key role in creating new phenotypes, and synthetic allopolyploids have helped us understand many genomic changes associated with polyploidization. With the success of RNAi technologies in down-regulating homoeologs in polyploids, demonstrated in sugarcane and Arabidopsis [178,179], it will be of interest to create polyploid crops that lack expression of genes involved in epigenetic regulation to investigate the involvement of epigenetics in homoeologous gene expression and their agronomical traits. Development of methods to synthesize allopolyploids and high-throughput sequencing technologies are expected to accelerate research on different allopolyploid plants. Combined analyses of phenotype, proteome, epigenome, transcriptome, and small RNAs in allopolyploid species will allow us to understand the mechanism of reproductive barriers, hybrid incompatibility, and allopolyploid gene regulation, and may provide insights into enhancing, manipulating, or controlling agronomical performance in allopolyploid crops.
